# Supplementary material for: MetaRibo-Seq measures translation in microbiomes
Source: Nat Commun. 2020 Jun 29;11:3268. doi: 10.1038/s41467-020-17081-z (PMC7324362; doi:10.1038/s41467-020-17081-z)
Supplement: Supplementary file 10 — Supplementary Data 7 [file 41467_2020_17081_MOESM10_ESM.zip › File2/Confidence_High_Taxonomy/22093_out.krona.html]

Javascript must be enabled to view this page.

members
magnitude
magnitudeUnassigned
count
unassigned
taxon
rank

22093\_out

2

2
2
superkingdom

phylum
976
1

200643
class
1

1
order
171549

1
family
815

816
genus
1

species

SRS893378\_contig\_number\_10590
46506
1

1
1239
phylum

class
186801
1

1
186802
order

1
31979
family

1
1485
genus


SRS1055049\_contig\_number\_contig-100\_22155.62290
1650661
species
1
